# Growth Promotion of *Salicornia bigelovii* by *Micromonospora chalcea* UAE1, an Endophytic 1-Aminocyclopropane-1-Carboxylic Acid Deaminase-Producing Actinobacterial Isolate

**DOI:** 10.3389/fmicb.2019.01694

**Published:** 2019-07-24

**Authors:** Khaled A. El-Tarabily, Abdulmajeed S. AlKhajeh, Mutamed M. Ayyash, Latifa H. Alnuaimi, Arjun Sham, Khaled Z. ElBaghdady, Saeed Tariq, Synan F. AbuQamar

**Affiliations:** ^1^Department of Biology, College of Science, United Arab Emirates University, Al Ain, United Arab Emirates; ^2^School of Veterinary and Life Sciences, Murdoch University, Murdoch, WA, Australia; ^3^Department of Food, Nutrition and Health Sciences, College of Food and Agriculture, United Arab Emirates University, Al Ain, United Arab Emirates; ^4^Department of Microbiology, Faculty of Science, Ain Shams University, Cairo, Egypt; ^5^Department of Anatomy, College of Medicine and Health Sciences, United Arab Emirates University, Al Ain, United Arab Emirates

**Keywords:** ACC deaminase, halophyte, endophytic actinobacteria, plant growth promotion, *Salicornia bigelovii*

## Abstract

*Salicornia bigelovii* is a promising halophytic crop for saline soils in semi-arid regions. This study was designed to characterize isolates of endophytic actinobacteria from *S. bigelovii* roots and evaluate the effects associated with plant growth promotion. Twenty-eight endophytic isolates obtained from surface-sterilized roots of *S. bigelovii* were initially selected based on their production of 1-aminocyclopropane-1-carboxylic acid (ACC) deaminase *in vitro* in a chemically defined medium. Application of *Micromonospora chalcea* UAE1, possessing the highest ACC deaminase activity, to *S. bigelovii* seedlings significantly enhanced the plant growth under gnotobiotic and greenhouse conditions. This was clear from the increases in the dry weight and length of both shoot and root, and seed yield compared to the non-ACC deaminase-producing isolate *Streptomyces violaceorectus*, or control treatment. The growth promotion was also supported by significant increases in the content of photosynthetic pigments and the levels of auxins, but significant decreases in the levels of ACC *in planta*. Under greenhouse conditions, *M. chalcea* recovered from inside the inoculated roots in all samplings (up to 12 weeks post inoculation), suggesting that the roots of healthy *S. bigelovii* are a suitable habitat for the endophytic actinobacterial isolates. Pure cultures of *M. chalcea* were not capable of producing auxins, gibberellic acid, cytokinins or polyamines *in vitro*. This indicates that the growth promotion is most likely to be due to the reduction of the endogenous levels of the stress hormone ethylene. Our findings suggest that growth and yields of *S. bigelovii* can be enhanced by the field application of the endophyte *M. chalcea* UAE1. This study is the first to report potential endophytic non-streptomycete actinobacteria to promote the growth of halophytic plants in semi-arid zones under greenhouse conditions.

## Introduction

*Salicornia* (glasswort) is a genus of annual, leafless, and fast-growing halophytic plant that can grow in coastal salt marshes and in inland salty habitats (Wiggins, 1980; Shepherd et al., 2005). In general, *Salicornia* spp. are widely managed in climates ranging from temperate to tropical (Ventura and Sagi, 2013). This succulent plant is used for human consumption and animal feed (Belal and Al-Dosari, 1999; Doncato and Costa, 2018); and its oilseeds are potential source for biofuel and biodiesel production (Bailis and Yu, 2012; Abideen et al., 2015). Thus, *Salicornia* spp. are promising crops for saltwater and seawater-irrigated agriculture in the coastal deserts of many countries including Eritrea (Zerai et al., 2010), Kuwait (Abdal, 2009), the United States (Glenn et al., 1991), and United Arab Emirates (UAE) (Al-Yamani et al., 2013; Shahid et al., 2013).

Soil salinity is a serious environmental stress that affects global agricultural productivity (Mayak et al., 2004). Saline soils are common in arid and semi-arid regions, where diversified species of halophytes dominate (English and Colmer, 2011). In response to high salinity stress, plants produce increased levels of ethylene (ET), which inhibits plant growth and development (Abeles et al., 1992; Glick, 2014). Several phases of plant growth are regulated by ET, although this hormone plays central roles in plant responses to biotic and abiotic stresses (Glick et al., 2007; Mengiste et al., 2010; Glick, 2014).

Bacteria are present not only on the root surface (rhizoplane) or in the rhizosphere, but can also be found endophytically within tissues of host plants (Sturz et al., 2000; Hardoim et al., 2015). By definition, endophytic bacteria can live within living plant tissues without causing any substantial harm to plants (Kado, 1992), and can be isolated from surface-sterilized plant tissue or extracted from inside the plant (Hallmann et al., 1997). Several studies have shown that the endophytic bacteria-plant interaction can be associated with beneficial effects to the host plant. Such benefits include, but not limited to, plant growth promotion (PGP), nitrogen (N) fixation, biological control against pathogens, induction of systemic resistance to plant pathogens, improvement of phytoremediation, and crop adaptation to environment challenges (Sturz et al., 2000; Rosenblueth and Martínez-Romero, 2006; Trujillo et al., 2015; Khare et al., 2018). In comparison with rhizospheric bacteria, endophytic bacteria can proliferate inside the plant tissue, interact closely with the host, face less competition for nutrients, and be more protected from environmental cues (Rosenblueth and Martínez-Romero, 2006; Khare et al., 2018). Therefore, this interaction has a potential in developing sustainable crop production (Carvalho et al., 2017; Khare et al., 2018).

Endophytic plant growth promoting bacteria (PGPB) may stimulate growth and increase yield in plants indirectly or directly. Indirect mechanisms of growth promotion may include production of iron (Fe)-sequestering siderophores or compounds with antimicrobial or antifungal activities; thus this serves to protect plants from soil-borne pathogens (Rosenblueth and Martínez-Romero, 2006; El-Tarabily et al., 2009; Khare et al., 2018). Direct mechanisms may involve the fixation of atmospheric N, synthesis of siderophores, solubilization of minerals such as phosphorus (P) or production of plant growth regulators (PGRs) such as auxins, cytokinins, gibberellins and polyamines which directly affect plant growth (Rosenblueth and Martínez-Romero, 2006; Glick, 2015; Qin et al., 2015; Trujillo et al., 2015; Olanrewaju et al., 2017; Khare et al., 2018). In addition, many PGPB have been investigated to stimulate plant growth through the activity of 1-aminocyclopropane-1-carboxylic acid (ACC) deaminase (Glick, 2014, 2015; Olanrewaju et al., 2017). This enzyme hydrolyzes ACC, which is the immediate biosynthetic precursor of the hormone ET in plant tissues, to ammonia and α-ketobutyrate. Inoculation of plants with ACC deaminase-producing PGPB lowers the levels of ACC, reduces the harmful effects of ET synthesized as a consequence of stressful conditions, and therefore promotes plant growth (Glick et al., 2007; Sarkar et al., 2018; Acuna et al., 2019). ACC deaminase-producing PGPB have been tested to mitigate the inhibitory effects of salinity stresses on plant growth and development (Sarkar et al., 2018; Acuna et al., 2019). Hence, the introduction of ACC deaminase-producing PGPB may dramatically increase the productivity of *Salicornia* crops.

Many bacteria have been isolated from the rhizosphere of *Salicornia* spp. (Bashan et al., 2000; Rueda-Puente et al., 2003, 2004; Mapelli et al., 2013) or from within living tissues of *Salicornia* spp. as endophytes (Jha et al., 2012; Zhao et al., 2016; Yamamoto et al., 2018). However, few studies have reported the use of rhizospheric and endophytic bacteria for growth promotion of *Salicornia* spp. under greenhouse and/or field conditions. Some studies have determined the potential of bacteria isolated from the rhizosphere of mangrove and *Salicornia* spp. (Bashan et al., 2000; Rueda-Puente et al., 2003, 2004) or roots of *Salicornia* spp. (Ozawa et al., 2007) to enhance *Salicornia* spp. growth and performance in saline soils under greenhouse conditions. These reports, however, have been attributed to growth enhancement only through N-fixation, albeit the use of PGPB which can produce PGRs or ACC deaminase. Bacteria isolated from *Salicornia* spp. roots and rhizosphere have the ability to produce indole-3-acetic acid (IAA) and ACC deaminase in chemically defined liquid media (Jha et al., 2012; Mapelli et al., 2013; Zhao et al., 2016). These isolates have been tested on seed germination and seedling performance of *Salicornia* spp. at different concentrations of NaCl *in vitro* (Jha et al., 2012). Zhao and colleagues (2016) have reported that the isolated ACC deaminase-producing endophytic bacteria from tissues of *Salicornia europaea* promoted the growth of *S. europaea* seedlings at 50–500 mM NaCl under axenic conditions. Up-to-date, no attempt has been made to test rhizospheric or endophytic actinobacteria for their potential to enhance *Salicornia* spp., growth irrigated with seawater in the greenhouse or the open field.

At present, there has been a considerable interest in the application of bacterial inoculants to promote the growth of halophytic forage and oilseed crops such as *Salicornia* spp. (Sáenz-Mata et al., 2016; Mesa-Marin et al., 2019). In the UAE, there is a great value in cultivating *Salicornia bigelovii* using the seawater irrigation system for animal feed (Al-Yamani et al., 2013; Shahid et al., 2013), aviation biofuel (Bresdin et al., 2016) and for possible forms of renewable energy (Jamil et al., 2016). The overall aim of this research was to promote the growth of *S. bigelovii* by endophytic PGP actinobacteria. The objectives of the present investigation were to: (i) isolate endophytic actinobacteria from *S. bigelovii* roots capable of producing ACC deaminase and determine their abilities to promote growth of *S. bigelovii* under gnotobiotic conditions; (ii) evaluate the most promising ACC deaminase-producing isolate that endophytically colonizes *S. bigelovii* roots; and (iii) determine the response of *S. bigelovii* to inoculation with endophytic actinobacteria under controlled greenhouse conditions, by evaluating plant growth, and endogenous levels of auxins and ACC in tissues.

## Materials and Methods

### Soil Characteristics and Plant Material

Grayish, pale yellowish sandy arenosol soil in which *S. bigelovii* is naturally found was collected from the coast of Al Rams (25° 52′ 44′′ N, 56° 1′ 25′′ E), Ras Al Khaimah, UAE. The chemical characteristics of the soil were: pH 8.18 (in 0.01 M CaCl_2_); electrical conductivity 5.81 dSm^-1^; and organic carbon 1.15%. These nutrients (mg kg soil^-1^) were detected: bicarbonate extractable potassium (K) and available P (265 and 8.3, respectively); total P (44), N (2.9 as nitrate; 5.3 as ammonium); sulfate (311) and oxalate extractable amorphous iron (382). Seeds of *S. bigelovii* Torr. were purchased from Scrops (Belgium).

In all experiments, healthy seeds were surface-sterilized by soaking in 70% ethyl alcohol (EtOH; Sigma-Aldrich Chemie GmbH, Germany) for 4 min and immersed in 1.05% NaOCl (20% Clorox) for 4 min. A 0.05 ml l^-1^ surfactant (Tween 20; Sigma-Aldrich) was used in all surface-sterilization procedures. Seeds were washed ten times with sterile distilled water and air-dried for 25 min.

### Isolation of Endophytic Actinobacteria From *S. bigelovii* Roots

Free draining plastic pots (23 cm in diameter) were filled with soil collected from the same area described above (section “Soil Characteristics and Plant Material”). Seeds of *S. bigelovii* were surface-sterilized (section “Soil Characteristics and Plant Material”) and sown in pots. Pots were maintained in a greenhouse at 25 ± 2°C (photosynthetic photon flux density of 700 μmol m^-2^ s^-1^) and relative humidity of 60 ± 5%. Pots were watered daily to container capacity with full strength seawater (salinity 40 ppt). The experiment was replicated five times with four plants in each replicate.

After 4 weeks, *S. bigelovii* seedlings were uprooted and transferred to the laboratory. To isolate endophytic actinobacteria, roots were cut from stems, rinsed in running tap water, and the fresh root weight was recorded. Roots were soaked in sterile phosphate-buffered saline solution (PBS; pH 7.0) for 10 min to prevent passive diffusion of sterilizing agents into the roots and to equilibrate osmotic pressure (Rennie et al., 1982).

For the surface-sterilization, roots were firstly exposed to propylene oxide (Sigma-Aldrich) vapor for 25 min (Sardi et al., 1992). Roots were soaked in 70% EtOH for 4 min and 1.05% NaOCl for 4 min; followed by rinsing ten times in PBS (Hallmann et al., 1997).

Sterility checks were carried out for each sample in order to verify no transmission of biological contamination into the root tissues during maceration. Briefly, root impressions were taken and 0.2 ml from the final rinse was plated out on tryptic soy agar plates (Lab M Ltd., United Kingdom) (McInroy and Kloepper, 1995; Sturz et al., 1998). A final 1-ml buffer from the final rinse solutions was transferred to 9 ml tryptic soy broth (Lab M Ltd.) incubated at 28 ± 2°C in dark. In a 4-day period, the absence of bacterial growth confirmed that the obtained actinobacteria were considered to be purely endophytic.

Roots were soaked in 100 ml of PBS using Omni-mixer (Omni International, United States) at 4,000 rpm for 20 min. The slurry was filtered using sterile filter papers, and the filtrate was serially diluted (10^-2^–10^-5^) in PBS (Hallmann et al., 1997). Aliquots (0.2 ml) were spread on starch nitrate agar (SNA) (Küster, 1959) made using seawater for the enumeration of the total populations of endophytic actinobacteria. Cooled sterile SNA (45°C) was amended with 50 μg ml^-1^ of each nystatin and cycloheximide (Sigma-Aldrich) to inhibit fungal growth. Plates were dried and then incubated for 7 days at 28 ± 2°C in dark. Three replicated plates were used for each dilution for each root sample. Population densities were expressed as log_10_ colony forming units (cfu) g^-1^ fresh root weight (Hallmann et al., 1997).

Following incubation, actinobacterial colonies were transferred on oatmeal agar plates amended with 0.1% yeast extract OMYEA (ISP medium 3) (Shirling and Gottlieb, 1966). Streptomycete actinobacteria (SA) and non-streptomycete actinobacteria (NSA) were identified based on cultural and morphological characteristics according to Cross (1989). Hyphae and spores of all isolates were stored in 20% glycerol (cryoprotectant) at -70°C (Wellington and Williams, 1977).

### Determination of ACC Deaminase Activity by Endophytic Actinobacterial Isolates

All endophytic actinobacterial isolates were screened for the production of ACC deaminase from ACC using N-free Dworkin and Foster’s salts minimal agar medium (DF) (Dworkin and Foster, 1958). The medium was amended with either 2 g (NH_4_)_2_SO_4_ (control) or 3 mM ACC (Sigma-Aldrich) as sole N source. The heat-labile ACC was filter-sterilized and the filtrate was added to the salt medium.

Five-day-old isolates grown on rich OMYEA were streaked in triplicates on DF plates supplemented with either (NH_4_)_2_SO_4_ or ACC. Plates were incubated for 7 days at 28 ± 2°C in dark. Isolates growing on DF+ACC plates were taken as indicators of the efficiency of selected isolates to utilize ACC and to produce ACC deaminase.

To determine the ACC deaminase activity, isolates were grown in starch nitrate broth (SNB) for 5 days at 28 ± 2°C in dark. Spores were then harvested by centrifugation, washed with 0.1 M Tris–HCl (pH 8.5), inoculated onto DF+ACC broth on a rotary shaker at 250 rpm for another 5 days at 28 ± 2°C in dark. The cells were collected, resuspended in 0.1 M Tris–HCl, and ruptured by three freezing/thawing cycles (Shah et al., 1998). The lysate was centrifuged at 80,000 × *g* for 1 h and the supernatant was assayed for ACC deaminase activity by monitoring the amount of α-ketobutyrate that was produced by the deamination of ACC as previously described (Honma and Shimomura, 1978). Protein concentrations were determined as previously described (Bradford, 1976). Six independent flasks for each isolate were analyzed.

### Assessment of PGP Parameters Under Gnotobiotic Conditions

Based on the quantifications of ACC deaminase (section “Determination of ACC Deaminase Activity by Endophytic Actinobacterial Isolates”), isolates displaying moderate-high ACC deaminase activity (low = 100; moderate = 100–200; high ≥ 200 nmol α-ketobutyrate mg^-1^ protein h^-1^) were selected for further experiments under gnotobiotic conditions in the presence or absence of 10 μM L-α-(aminoethoxyvinyl)-glycine (AVG; Sigma-Aldrich) (ET inhibitor) or 10 μM 2-chloroethylphosphoric acid (ethephon; Sigma-Aldrich) (ET generator) using sterilized sand (Chanway et al., 1991). An ACC deaminase-non-producing endophytic isolate was also included in all experiments.

Sand was acid-washed in 1:1 (w/v) sand:6M HCl, rinsed with deionized water and autoclaved. Glass tubes (300 × 35 mm in diameter) filled with the autoclaved sand were moistened with seawater and autoclaved. To each tube, filter-sterilized nutrient solution (65 ml; Arsac et al., 1990) was added as a single application after planting the seedlings (Frankenberger et al., 1990). Seedlings were inoculated with the actinobacterial suspensions using the pruned-root dip method (Musson et al., 1995). Briefly, roots of pre-germinated *S. bigelovii* seedlings (4 days old) were trimmed to uptake of the actinobacterial inoculum (Musson et al., 1995; Bressan and Borges, 2004). Seedlings were placed in sterile plastic cups for 3 h at 25°C with their roots in contact with the inoculum suspension of each isolate (10^8^ cfu ml^-1^). Control seedlings were treated with autoclaved SNB. All seedlings were planted in the tubes under aseptic conditions, irrigated every other day with full strength filter-sterilized seawater and maintained in a growth chamber under a 16/8-h day (180–200 μmol m^-2^ s^-1^ fluorescent light)/dark and 25/20°C light/dark temperature cycle. After 4 weeks of transplantation, plants were harvested, washed and separated into roots and shoots. The weights and lengths of shoots and roots were measured to determine growth of *S. bigelovii*. Each treatment was independently replicated eight times with one seedling in each replicate.

### PGP Activities by Selected ACC Deaminase Producing Endophytic Isolates

The five ACC deaminase-producing isolates in addition to the ACC deaminase-non-producing isolate were also examined *in vitro* for their abilities to produce other PGRs, including auxins, i.e., IAA and indole-3-pyruvic acid (IPYA), gibberellic acid (GA3), and cytokinins, i.e., isopentenyl adenine (iPa), isopentenyl adenoside (iPA), and zeatin (Z). Isolates were grown in 50 ml glucose peptone broth (di Menna, 1957) supplemented with 5 ml of 5% L-tryptophan (Sigma-Aldrich) to detect auxins, and on medium developed by Strzelczyk and Pokojska-Burdziej (1984) to detect GA3 and cytokinins using high performance liquid chromatography (HPLC) (Tien et al., 1979).

Qualitative production of polyamines was carried out using the modified Moeller’s decarboxylase agar medium (MDAM) supplemented with 2 g l^-1^ of L-arginine-monohydrochloride (Sigma-Aldrich) and 0.02 g l^-1^ phenol red (Sigma-Aldrich) as a pH dye indicator (Arena and Manca de Nadra, 2001). Five-day-old isolates grown on OMYEA were streaked on MDAM plates with or without arginine (control), and incubated for 4 days at 28 ± 2°C in dark. Growth of the decarboxylating isolates was identified according to the presence of a dark red halo around and beneath the colonies.

Phosphate solubilizing ability was analyzed by the colorimetric procedure using molybdophosphoric acid blue complex (Murphy and Riley, 1962). The efficiency of the strains to solubilize P was evident by the pH drop and the amount of released soluble P. Activities of N-fixation were examined by the acetylene-reduction assay using Varian 6000 gas chromatogram (Varian Instrument Group, United States), described by Holguin et al. (1992). Ammonia production was determined using Nessler’s reagent (Dye, 1962). To determine the production of siderophores, plates of chrome azurol S (CAS) agar (Schwyn and Neilands, 1987), were inoculated with the isolates and incubated for 3 days at 28 ± 2°C in dark. Development of yellow-orange halo zone around the colony was considered positive for siderophore production. For all these tests, six independent replicates for each strain were used.

### Determination of Actinobacterial Tolerance to Different Concentrations of NaCl

Tolerance to salt stress was assessed by growing the selected six isolates on SNA medium supplied with different NaCl concentrations (0, 5, 10, 15, 20, and 40 g l^-1^ medium). The isolates were streaked in triplicates on plates and incubated for 7 days at 28 ± 2°C in the dark (Williams et al., 1972). Growth and sporulation of the isolates on SNA amended with 40 g NaCl l^-1^ indicated the efficiency of isolates to tolerate high concentration of NaCl.

### Identification and Construction of Phylogenetic Tree of Selected Endophytic Actinobacteria

The most potent ACC deaminase-producing (#11) and -non-producing (#22) isolates were identified based on 16*S* rRNA gene sequence analysis by the Deutsche Sammlung von Mikroorganismen und Zellkulturen GmbH, (DSMZ), Braunschweig, Germany, using 900R (5′-CCGTCAATTCATTTGAGTTT-3′); 357F (5′-TACGGGAGGCAGCAG-3′) and 800F (5′-ATTAGATACCCTGGTAG-3′) primers (Rainey et al., 1996; Saeed et al., 2017; Kamil et al., 2018). To predict the species level characterization of isolates, we constructed a phylogenetic tree using the maximum likelihood method implemented in Molecular Evolutionary Genetics Analysis 7.0 (MEGA7) software (Kumar et al., 2016) after multiple alignments of the data by CLUSTAL-X (Thompson et al., 1997). Bootstrap values were calculated based on 1000 resamplings.

In order to determine the morphology of the spore chains and surface, scanning electron microscopy (SEM) was carried out for the two isolates using Philips XL-30 SEM (FEI Co., Netherlands).

### Production of Inoculum and Estimation of Internal Root Colonization in the Greenhouse

Rifampicin-resistant mutants of the two isolates were prepared (Misaghi and Donndelinger, 1990). Mutants were selected on SNA medium supplemented with 100 μg ml^-1^ of rifampicin (Sigma-Aldrich), and were tested for their stability by repeated transfers to medium without rifampicin followed by a transfer to medium supplemented with rifampicin. Resistant mutants were compared with their corresponding wild type strains according to their ability to produce ACC deaminase. None of these mutants differed morphologically from their parental strains, and the mutants had growth rates and ACC deaminase-producing activities comparable to those in the parental strains. To produce inoculum for the greenhouse experiments, aliquots (4 ml) of 20% glycerol suspension of the two endophytes were inoculated into 250-ml SNB and shaken on a rotary shaker at 250 rpm for 5 days. Cells were harvested by centrifugation (12,000 × *g* at 20°C for 15 min) and the resultant pellet was suspended in 10 ml PBS and re-centrifuged. A dilution series of each suspension was made in PBS of which 0.1 ml of each 10^-4^, 10^-5^, and 10^-6^ dilutions was spread on SNA. Plates were incubated for 5 days to a final concentration of approximately 10^8^ cfu ml^-1^ of each isolate to be used as an inoculum.

To assess the internal root colonization, we used the pruned-root dip method to inoculate *S. bigelovii* seedlings with each isolate as previously discussed (section “Assessment of PGP Parameters Under Gnotobiotic Conditions”). Free-draining pots, filled with sieved soil collected from the same area described in section “Soil Characteristics and Plant Material,” were placed in an evaporative-cooled greenhouse, and seedlings were watered daily to container capacity with full strength seawater. Every week after planting (weeks 1–12), roots were sampled and the population densities of the isolates were determined on SNA amended with rifampicin (100 μg ml^-1^). Each treatment was replicated four times with four plants per replicate for each sampling.

For light microscopy (LM) and transmission electron microscopy (TEM), roots of *S. bigelovii* (4-week-old seedlings) previously inoculated with the highest ACC deaminase activity isolate, were fixed in 2% glutaraldehyde in 0.17 M phosphate buffer (pH 7.2) under vacuum for 24 h at 25°C, followed by four cycles of washing in the same buffer. Samples were post-fixed in 1% osmium tetroxide in 0.17 M phosphate buffer for 2 h, rinsed with distilled water, dehydrated with ethanol solutions, embedded in epoxy resin (Epon 812, Agar Scientific, United Kingdom) and polymerized for 24 h at 60°C (Millonig, 1976). Semi-thin transverse sections (0.5-μm) were cut and stained with 0.1% toluidine blue for observation under the LM. Sections were examined using Olympus BH-2 microscope (Olympus Optical Co. Ltd., Japan). For TEM, ultra-thin sections (90-nm) were stained with uranyl acetate and lead citrate, and examined with a Philips CM10 TEM (FEI Co., Netherlands) operating at 80 kV.

### Evaluation of Growth Promotion of *S. bigelovii* Under Greenhouse Conditions

The effect of isolates on *S. bigelovii* growth was further tested *in vivo* under greenhouse conditions. Soil was collected from the same location as described above. Free-draining pots of non-inoculated- and inoculated-seedlings with the isolates (section “Assessment of PGP Parameters Under Gnotobiotic Conditions”), using the pruned-root dip method, were planted. In general, there were a total of three treatments as the following: (1) seedlings inoculated with autoclaved SNB medium only (control); (2) seedlings inoculated with the ACC deaminase-non-producing isolate; and (3) seedlings inoculated with the ACC deaminase-producing isolate. Each treatment was replicated eight times (four plants/replicate) in a randomized complete block design (RCBD). Pots were placed in a greenhouse and seedlings were watered daily to container capacity with full strength seawater. Plant growth was weekly followed by recording the fresh and dry weight and length of shoots and roots at 12 weeks post transplantation (wpt) of seedlings; and seed dry weight at the time of harvest (20 wpt).

### Extraction of Photosynthetic Pigments, Auxins, and ACC From *S. bigelovii*

The levels of chlorophyll (chl *a* and chl *b*(and carotenoids in the succulent stems were determined (Davies, 1965; Holden, 1965). Eight samples were analyzed for each treatment. The endogenous IAA and IPYA were extracted according to Guinn et al. (1986) and the HPLC parameters were applied (Tien et al., 1979). The extraction of endogenous ACC was also carried out using a method described by Lizada and Yang (1979). Derivatization of ACC was done by adding phenylisothiocyanate (Sigma-Aldrich) and the HPLC chromatograms were produced as previously described (Lanneluc-Sanson et al., 1986). Four independent samples were analyzed for IAA, IPYA, and ACC. All extraction procedures were conducted on plants at 12 wpt.

### Statistical Analyses

For all experiment, treatments were arranged in a RCBD. Repeated gnotobiotic and greenhouse experiments showed similar patterns with no significant differences between the experiments. Hence, the data from the repeated experiments were combined and analyzed. Data of bacterial population densities were transformed into log_10_ cfu g^-1^ fresh root weight. Data were subjected to analysis of variance (ANOVA) and treatment means were compared using Fisher’s Protected LSD Test at *P* = 0.05. For all statistical analyses, SAS Software version 9 was used (SAS Institute, 2002).

## Results

### Screening of Endophytic Actinobacteria for the Production of ACC Deaminase Under Gnotobioitic Conditions

We first determined the population of the endophytic actinobacteria in *S. bigelovii* roots which was 4.72 ± SE 0.95 log_10_ cfu g^-1^ fresh root weight. No contamination in the sterility checks was found, suggesting that the surface-sterilization procedures were adequate. A total of 28 different endophytic actinobacteria were isolated from the root triturate on SNA plates (Supplementary Figure S1), of which 15 (53.6%) SA and 13 (46.4%) NSA isolates were identified based on their cultural characteristics. The SA and NSA (e.g., *Actinoplanes, Micromonospora, Rhodococcus*, and *Nocardia* spp.) formed discrete colonies and were easy to enumerate and isolate. Out of the 28 obtained isolates, only 11 (39.3%) were able to grow and sporulate on DF+ACC agar (Table 1). The rest of isolates grew only on DF supplemented with (NH_4_)_2_SO_4_ (control), but not on DF+ACC agar. This suggests that most likely the latter group of isolates may not have any ACC deaminase activity and consequently were eliminated. We also noticed that the production of ACC deaminase varied significantly (*P* < 0.05) among the tested isolates (Table 1). Quantitative assays of ACC deaminase revealed that isolates #2, #7, #11, #18, and #26 produced moderate to high levels of ACC deaminase activity and were considered as producers of ACC deaminase. These promising isolates were selected for further analyses. In addition, the actinobacterial strain #22 was selected as an ACC deaminase-non-producing isolate.

**Table 1:** Production of 1-aminocyclopropane-1-carboxylic acid (ACC) deaminase by actinobacterial isolates grown in Dworkin and Foster’s salts minimal broth medium (DF) amended with ACC after 5 days of incubation at 28 ± 2°C.

Isolate	ACC deaminase activity (nmol
	α-ketobutyrate mg^-1^ protein h^-1^)^b^
#2	147.28 ± 8.77 *d*
#3	46.17 ± 3.52 *ef*
#7	188.14 ± 6.58 *c*
#9	36.94 ± 6.37 *ef*
#11^a^	457.27 ± 16.38 *a*
#14	52.07 ± 8.03 *ef*
#18	263.55 ± 19.56 *b*
#20	34.53 ± 3.46 *f*
#22^a^	0.00 ± 0.00 *g*
#23	42.41 ± 8.29 *ef*
#25	64.75 ± 7.70 *e*
#26	153.11 ± 15.71 *d*

*^*a*^Isolates #11 and #22 represent *Micromonospora chalcea* UAE1 and *Streptomyces violaceorectus* UAE1, respectively. ^*b*^Values are means of 12 replicates ± SE for each sampling from two independent experiments. Mean values followed by different letters are significantly (*P* < 0.05) different from each other according to Fisher’s Protected LSD Test.*

The five actinobacterial isolates, with moderate to high levels of ACC deaminase activity, were screened for their growth promoting activity in *S. bigelovii* under gnotobiotic conditions. In general, inoculation with these isolates had significant (*P* < 0.05) increase in root elongation, shoot length and seedling (root and shoot) fresh weight of *S. bigelovii* compared to isolate #22 or control treatment (Table 2). Thus, the endophytic isolates showed variable effects on root and shoot growth. Inoculation with isolate #11 resulted in maximum increase in root and shoot length, which was 57.7 and 45.3% higher than the uninoculated plants, respectively. In addition, the same endophytic isolate significantly (*P* < 0.05) increased root (72.5%) and shoot (60.1%) fresh weights, compared to non-inoculated treatments (Table 2). This superiority of isolate #11 was followed by isolates #18 and #7, followed by isolates #2 and #26. All tested growth promotion characteristics using the endophytic ACC deaminase-non-producing isolate #22 were comparable to those of non-inoculated *S. bigelovii* plants (Table 2).

**Table 2:** Comparisons on the effect of ACC deaminase-producing endophytic actinobacterial isolates on *Salicornia bigelovii* growth parameters in the presence of AVG (ET inhibitor) or ethephon (ET generator) under gnotobiotic conditions.

Isolate	Treatment	Length (cm)^b^		Fresh weight (g)^b^	
		Root	Shoot	Root	Shoot
Control (MgSO_4_)	–	7.42 ± 0.56 *g*	12.74 ± 0.26 *g*	3.01 ± 0.05 *i*	10.51 1.06 *i*
	AVG	9.37 ± 0.31 *f*	15.28 ± 0.18 *f*	4.72 ± 0.08 *h*	12.53 ± 0.25 *h*
	Ethephon	2.56 ± 0.18 *j*	5.98 ± 0.09 *k*	0.38 ± 0.02 *l*	2.25 ± 0.24 *m*
#2	–	11.49 ± 0.74 *e*	17.18 ± 0.35 *e*	5.52 ± 0.09 *g*	15.81 ± 0.43 *g*
	AVG	11.30 ± 0.36 *e*	17.80 ± 0.28 *e*	5.45 ± 0.09 *g*	14.56 ± 0.79 *g*
	Ethephon	4.39 ± 0.21 *i*	7.59 ± 0.20 *j*	1.28 ± 0.11 *k*	5.35 ± 0.53 *l*
#7	–	12.65 ± 0.15 *d*	19.10 ± 0.47 *d*	6.47 ± 0.35 *f*	18.06 ± 0.17 *f*
	AVG	13.61 ± 0.25 *d*	19.65 ± 0.73 *d*	8.15 ± 0.56 *e*	20.06 ± 0.55 *e*
	Ethephon	3.96 ± 0.44 *i*	8.88 ± 0.20 *i*	1.58 ± 0.09 *kj*	5.08 ± 0.59 *l*
#11^a^	–	17.55 ± 0.46 *b*	23.29 ± 0.45 *b*	10.96 ± 0.44 *b*	26.35 ± 0.49 *b*
	AVG	18.80 ± 0.26 *a*	25.71 ± 0.66 *a*	13.01 ± 0.45 *a*	29.16 ± 0.39 *a*
	Ethephon	6.11 ± 0.03 *h*	10.51 ± 0.32 *h*	2.08 ± 0.03 *j*	8.25 ± 0.39 *j*
#18	–	15.74 ± 0.61 *c*	21.38 ± 0.53 *c*	9.08 ± 0.32 *d*	22.88 ± 0.19 *d*
	AVG	15.55 ± 0.53 *c*	21.42 ± 0.32 *c*	9.98 ± 0.33 *c*	24.61 ± 0.46 *c*
	Ethephon	5.68 ± 0.11 *h*	10.37 ± 0.47 *h*	1.79 ± 0.02 *kj*	6.76 ± 0.22 *k*
#22^a^	–	7.83 ± 0.37 *g*	12.09 ± 0.49 *g*	3.12 ± 0.03 *i*	10.03 ± 0.28 *i*
	AVG	9.52 ± 0.29 *f*	14.84 ± 0.31 *f*	4.47 ± 0.07 *h*	12.59 ± 0.47 *h*
	Ethephon	2.60 ± 0.33 *j*	5.78 ± 0.32 *k*	0.57 ± 0.05 *l*	3.45 ± 0.46 *m*
#26	–	10.91 ± 0.46 *e*	16.94 ± 0.41 *e*	5.52 ± 0.08 *g*	15.10 ± 0.29 *g*
	AVG	11.13 ± 0.45 *e*	17.71 ± 0.19 *e*	5.64 ± 0.18 *g*	14.94 ± 0.62 *g*
	Ethephon	4.19 ± 0.21 *i*	8.40 ± 0.26 *ji*	1.37 ± 0.12 *k*	4.98 ± 0.38 *l*

*^*a*^Isolates #11 and #22 represent *Micromonospora chalcea* UAE1 and *Streptomyces violaceorectus* UAE1, respectively. *S. violaceorectus* UAE1 was used as an ACC deaminase-non-producing actinobacterial isolate. ^*b*^Plants were harvested after 4 weeks. Values are means of 16 replicates for each treatment from two independent experiments. Mean values followed by different letters within a column are significantly (*P* < 0.05) different from each other according to Fisher’s Protected LSD Test. ACC, 1-aminocyclopropane-1-carboxylic acid; ET, ethylene; AVG, L-α-(aminoethoxyvinyl)-glycine; ethephon, 2-chloroethylphosphoric acid; –, no AVG or ethephon treatment.*

In general, the application of AVG (alone or in combination with any of the isolates) enhanced growth of *S. bigelovii* under gnotobiotic conditions (Table 2). Our results demonstrated that the ACC deaminase-producing isolates with the addition of AVG significantly (*P* < 0.05) increased the length and fresh weight of shoot and root of *S. bigelovii*, with isolate #11 showing the greatest increase in all the tested parameters. Ethephon application, however, significantly (*P* < 0.05) inhibited growth of *S. bigelovii* (Table 2). The presence of isolate #11 within *S. bigelovii* roots was found to be superior among all other isolates in promoting the growth of *S. bigelovii*, even when combined with ethephon (Table 2). Eventhough the five ACC deaminase-producing isolates significantly promoted shoot and root growth, isolate #11 was the most promising ACC deaminase activity candidate and the most inducing growth response of *S. bigelovii* under gnotobiotic conditions, which was further chosen for the greenhouse experiments.

### Evaluation of PGP Activities by ACC Deaminase Producing Endophytic Isolates

The endophytic actinobacteria were tested for their potential to produce auxins, gibberellic acid and cytokinins *in vitro*. Our results showed that there was no detection of auxins (IAA and IPYA), GA3 or Z in the culture extracts of the ACC deaminase-producing or -non-producing isolates (Table 3). In contrast to the cytokinin Z, some isolates produced small amounts of other cytokinins, iPa and iPA. For example, the ACC deaminase producers #2, #7, and #26 yielded 1.15, 0.75, and 1.77 μg ml^-1^ iPa, respectively. Similarly, the same isolates produced 0.85, 0.45, and 1.22 μg ml^-1^ iPA (Table 3). Thus, isolates #11 and #18 did not produce any PGRs.

**Table 3:** *In vitro* production of plant growth regulators, siderophores, nitrogenase enzyme, and ammonia, and the ability for phosphorus solubilization and tolerance to NaCl, by the five ACC deaminase-producing actinobacteria isolated from *Salicornia bigelovii* roots.

Activity	Isolate^a^					
	#2	#7	#11^b^	#18	#22^b^	#26
Indole-3-acetic acid (IAA)	–	–	–	–	–	–
Indole-3-pyruvic acid (IPYA)	–	–	–	–	–	–
Gibberellic acid (GA_3_)	–	–	–	–	–	–
Isopentenyl adenine (iPa)	+	+	–	–	–	+
isopentenyl adenoside (iPA)	+	+	–	–	–	+
Zeatin (Z)	–	–	–	–	–	–
Production of polyamines	+	–	–	–	–	+
Production of siderophores	+	+	–	+	–	–
Production of nitrogenase	–	–	–	–	–	–
Production of ammonia	–	–	–	–	–	–
Solubilization of phosphorus	–	–	–	–	–	–
Tolerance to NaCl	+++	+++	+++	+++	+++	+++

*^*a*^Data were from six independent replicates. ^*b*^Isolates #11 and #22 represent *Micromonospora chalcea* UAE1 and *Streptomyces violaceorectus* UAE1, respectively. *S. violaceorectus* UAE1 was used as an ACC deaminase-non-producing actinobacterial isolate. +, producing; -, not producing; +++, highly tolerant to NaCl.*

Because polyamines and siderophores are known for their essential roles in plant growth and development (Neilands, 1995; Walden et al., 1997), we evaluated the production of these PGP agents *in vitro*. The production of polyamines was only detected by isolates #2 and #26 (Table 3) as the dark red halo around and beneath the colonies appeared on MDAM medium. The endophytic actinobacteria #2, #7, and #18 (but not #11 or #26) that were considered as isolates with ACC deaminase activity were also involved in the production of siderophores (Table 3). Moreover, the five producing ACC deaminase were found to be neither P-solubilizing nor N-fixing isolates. Similarly, none of the isolates produced ammonia (Table 3). In all *in vitro* assays, the ACC deaminase-non-producing isolate #22 was not associated with PGP activities. It is noteworthy to mention that although isolate #11 was not active in any of the *in vitro* PGP activities (Table 3), it showed the highest activity of ACC deaminase among all tested isolates (Table 1). This suggests that isolate #11 is solely dependent on the production of ACC deaminase to promote plant growth.

In addition, the five ACC deaminase-producing isolates and the ACC deaminase-non-producing isolate grew very well and sporulated heavily on SNA medium amended with 0, 5, 10, 15, 20, and 40 g NaCl l^-1^ medium. All these isolates showed strong tolerance to high concentrations of NaCl (Table 3). This indicates that these endophytes can potentially be considered as halotolerant isolates.

### Identification and Characterization of the Endophytic Actinobacterial Isolates

**FIGURE 1:**
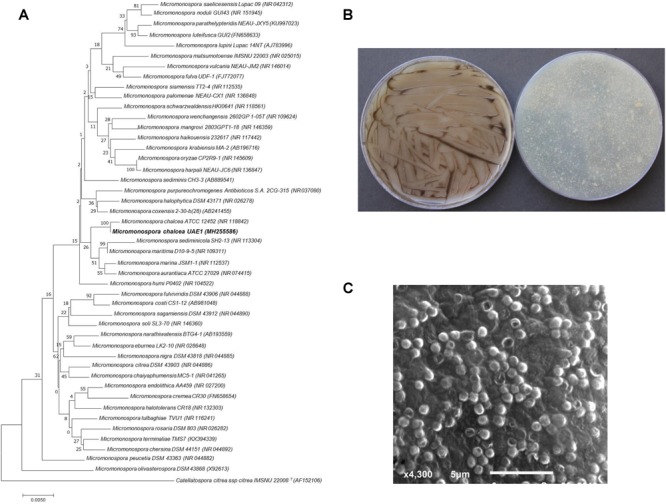
Taxonomic determination of *Micromonospora chalcea* UAE1, based on phylogenetic, cultural, and morphological characteristics. **(A)** The tree showing the phylogenetic relationships between *M. chalcea* UAE1 (*MH255586*; 1,480 bp) and other members of *Micromonospora* spp. on the basis of 16*S* rRNA sequences. **(B)** substrate mycelia without the formation of aerial mycelium (left) and the color of the substrate mycelia (right) growing on ISP medium 3 supplemented with yeast extract, and **(C)** scanning electron micrograph (4,000X) of the single oval to spherical smooth-surfaced spores of the strain of *M. chalcea* UAE1. In **(A)** numbers at nodes indicate percentage levels of bootstrap support based on a maximum likelihood analysis of 1,000 resampled datasets. Bar, 0.005 substitutions per site. *Catellatospora citrea* spp. *citrea* IMSNU 22008 (AF152106) was used as an outgroup. GenBank accession numbers are given in parentheses.

Based on the results obtained from ACC deaminase production and the growth promotion of *S. bigelovii* under gnotobiotic experiments, the most promising ACC deaminase-producing isolate (#11) and the ACC deaminase-non-producing isolate (#22) were further analyzed. The selected strains were identified based on the 16S rRNA gene sequence analysis. The 16S rRNA gene sequences of isolate #11 and #22 deposited in NCBI Genbank with accession number MH255586 and MH255588, respectively, were compared with that of other actinobacteria. The phylogenetic analysis of both isolates was constructed based on maximum likelihood method with 1000 bootstrap sampling. The 16S rRNA gene of isolates #11 and #22 were compared with sequences in the GenBank database, which showed that these endophytic actinobacterial candidates were a NSA sp. for isolate #11 (ACC deaminase-producing isolate) and SA sp. for isolate #22 (ACC deaminase-non-producing isolate). Isolate #11 showed above 99.7% similarity to *M. chalcea* (NR118842), although the remaining isolates of *Micromonospora* spp. showed less than 99.4% similarities (Figure 1A). The phylogenetic analysis of isolate #22 showed 100% similarity to *S. violacerorectus* (AB184314); while the rest showed < 99.8% similarity with this specific strain (Supplementary Figure S2). This may suggest that isolate #11 most probably be *M. chalcea*; while isolate #22 could be *S. violacerorectus*; thus a more reliable identification of these isolates can be obtained.

We further confirmed the identity of the ACC deaminase-producing and –non-producing endophytic actinobacteria. After 7 days of incubation at 28°C on ISP medium 3, pure cultures of isolate #11 developed raised, folded typical reddish-orange, turning dark brown colonies and eventually became black with sporulation (Figure 1B). This isolate produced substrate mycelium only with no formation of aerial mycelium. Using SEM, spores (0.8–1.0 μm in diameter) were found to be sessile, single, oval to spherical with smooth walls, and randomly appearing throughout the substrate mycelium (Figure 1C). Based on the phylogenetic, cultural, and morphological analyses, isolate #11 is to be *Micromonospora chalcea* (Foulerton, 1905), Ørskov, 1923, Strain UAE1. On the other hand, pure cultures of isolate #22 produced light grayish reddish aerial mycelium with dark purplish red substrate mycelial growth on ISP medium 3 after 7 days of incubation (Supplementary Figure S2). The isolate secreted reverse pinkish brown diffusible pigments, which was a pH indicator, in ISP medium 3. According to our observations, the configuration of the spore chains of isolate #22 belonged to rectiflexibiles (straight to flexuous chains) section (Supplementary Figure S2). Mature spore chains (>10 spores per chain) were generally long and straight, with smooth spore surface (Supplementary Figure S2). Our data suggest that isolate #22 can be recognized as *Streptomyces violaceorectus* (Ryabova and Preobrazhenskaya in Gauze et al., 1957; Pridham et al., 1958) Strain UAE1.

### Estimation of Internal Root Colonization by the Endophytic Actinobacterial Isolates

**FIGURE 2:**
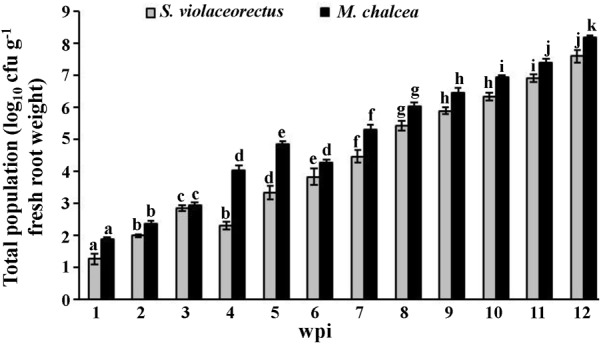
Total population of *Micromonospora chalcea* UAE1 and *Streptomyces violaceorectus* UAE1 in *Salicornia bigelovii* roots. The tested endophytic actinobacterial populations in the roots of *S. bigelovii* grown under greenhouse conditions were sampled at different time points. Values are means of eight replicates ± SE for each sampling from two independent experiments. Mean values followed by different letters are significantly (*P* < 0.05) different from each of the total populations of each strain according to Fisher’s Protected LSD Test. wpi, weeks post inoculation.

*Micromonospora chalcea* and *S. violaceorectus* were isolated from the surface-sterilized roots of *S. bigelovii* as endophytic strains. The two isolates were isolated from inoculated roots of *S. bigelovii* at all samplings on a weekly basis and maintained their endophytic colonizing abilities (Figure 2). Except at the third week, the total population of *M. chalcea* was significantly (*P* < 0.05) higher than that of *S. violaceorectus* in all tested samplings until week 12 (Figure 2). There was an increase in population densities of *M. chalcea* from week 1 to week 5, followed by a significant (*P* < 0.05) decrease in week 6. Between week 7 to 12, there was a significant (*P* < 0.05) increase in the frequency of recovery of *M. chalcea* within the roots of *S. bigelovii*. An initial increase in the population densities of *S. violaceorectus* was observed between weeks 1 to 3 (Figure 2); however, there was a significant (*P* < 0.05) drop in the total population at week 4. In later weeks, the population densities of *S. violaceorectus* steadily increased up to week 12 (Figure 2).

**FIGURE 3:**
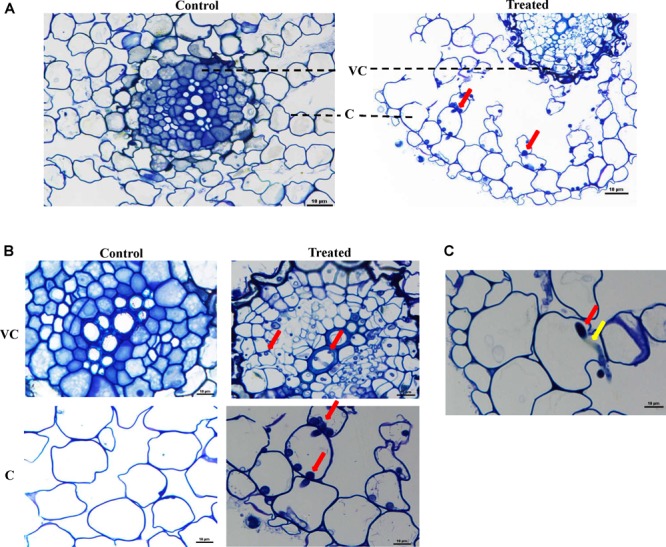
*Micromonospora chalcea* UAE1 colonizes the root tissues of *Salicornia bigelovii.* Light micrograph of semi-thin sections of a 2-week-old *S. bigelovii* root **(A)** not inoculated (control; left) or inoculated with *M. chalcea* UAE1 (treated; right) (400×); **(B)** close-up views of vascular cambium and cortex of *S. bigelovii* root inoculated with *M. chalcea* UAE1 (1000×); and **(C)** mycelial growth of *M. chalcea* UAE1 carrying spores penetrating *S. bigelovii* root cortex cells (1000×). In **(A–C)**, all sections were stained with 0.1% toluidine blue showing the distribution of spores (red arrows) and substrate mycelium (yellow arrows) within the roots cells of *S. bigelovii*. Bars: 10 μm. VC, vascular cambium; C, cortex.

**FIGURE 4:**
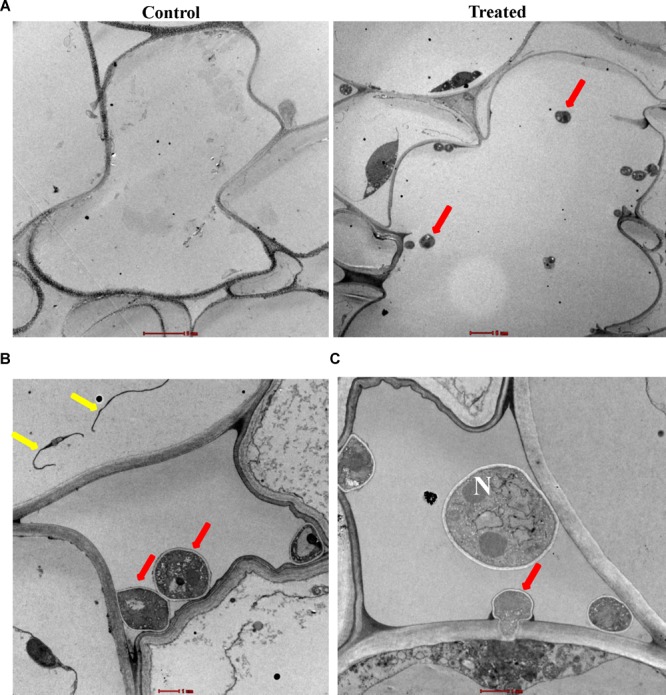
Inter- and intra-cellular colonization of *Salicornia bigelovii* roots by *Micromonospora chalcea* UAE1. Transmission electron micrograph of ultra-thin sections of a 2-week-old *S. bigelovii* root of **(A)** non-inoculated (control; left, 2550×) and inoculated with *M. chalcea* UAE1 (treated; right; 1250×); **(B)** spores and substrate mycelium of *M. chalcea* UAE1 within the cortex (6000×); and **(C)** penetration of *M. chalcea* UAE1 of a neighboring root cortical cell (8200×). In **(B,C)**, all sections were stained with uranyl acetate and lead citrate showing the distribution of spores (red arrows) and substrate mycelium (yellow arrows). Bars: **(A)** 5 μm; and **(B,C)** 1 μm. N, nucleus.

In LM studies on *S. bigelovii*-inoculated roots at 2 weeks post inoculation (wpi), the spores of *M. chalcea* UAE1 were abundantly present within the parenchyma cells of the cortex as well as in the xylem (Figure 3A,B). Mycelial growth carrying the spores of *M. chalcea* UAE1 within the cortical cells of *S. bigelovii* roots was also detected (Figure 3C). TEM studies further confirmed the presence of the endophytic actinobacterial strain (Figure 4A) along with the spores and substrate mycelium of *M. chalcea* UAE1 within the cortex (Figure 4B). Spores of *M. chalcea* UAE1 successfully penetrated the neighboring living root cortical cells (Figure 4C). We also figured out that some of the spores belonging to *M. chalcea* UAE1 were found to be in the process of germination (Figure 4B) and the formation of germ tubes (Figure 3C, 4B). Overall, our data suggest that *M. chalcea* UAE1 is an endophytic actinobaterial isolate that lives inside the root tissues of *S. bigelovii*.

### Assessment of *S. bigelovii* Growth Promotion Under Greenhouse Conditions

**FIGURE 5:**
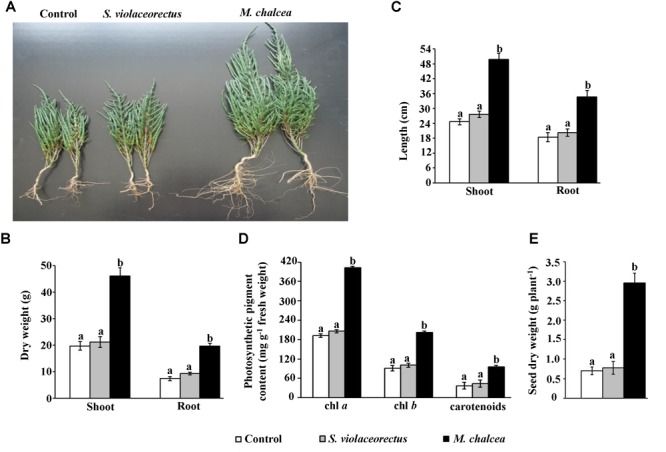
Effect of application of *Micromonospora chalcea* UAE1 on *Salicornia bigelovii* tissues. Effect of autoclaved starch nitrate broth medium (control; left), the ACC deaminase-non-producing isolate *Streptomyces violaceorectus* UAE1 (isolate #22, middle) or the ACC deaminase-producing *M. chalcea* UAE1 (isolate #11, right) inoculations on the **(A)** shoot and root formation of *S. bigelovii*. Measurements of the shoot and root **(B)** dry weight, **(C)** length, and **(D)** contents of chlorophyll *a* (chl *a*), chl *b* and carotenoids of *S. bigelovii* at 12 wpt. **(E)** Dry weight of *S. bigelovii* seeds harvested at 20 wpt. Seedlings growing in soil under evaporative-cooled greenhouse conditions, were inoculated using the pruned-root dip method. In **(B–E)**, values are means of 16 replicates ± SE for each sampling from two independent experiments. Mean values followed by different letters are significantly (*P* < 0.05) different from each other according to Fisher’s Protected LSD Test. wpt, weeks post transplantation.

In order to determine the effect of the ACC deaminase-producing isolate as a PGP candidate, we treated *S. bigelovii* seedlings with *M. chalcea* UAE1, *S. violaceorectus* or control (no *M. chalcea* or *S. violaceorectus*) treatments. In general, *M. chalcea* or *S. violaceorectus* inoculant did not produce any harmful effect on *S. bigelovii* (Figure 5A). The application of the ACC deaminase-producing isolate (isolate #11; *M. chalcea*) significantly (*P* < 0.05) promoted the growth and development of *S. bigelovii* in the greenhouse (Figure 5A). In this treatment, we noticed significant (*P* < 0.05) increases in the dry weight (Figure 5B) and length (Figure 5C) of roots and shoots, in comparison to the *S. bigelovii* treated with the *S. violaceorectus* or control treatment after 12 weeks. The application of *M. chalcea* induced the length of shoot and root by approximately 50% compared to non-inoculated plants (Figure 5C). Likewise, *S. bigelovii*-treated plants with the ACC deaminase-producing isolate had 57.2 and 62.5% greater dry weight of shoot and root, respectively, than that in non-inoculated plants (Figure 5B). On the other hand, there were no significant differences in the root or shoot growth features of *S. bigelovii* inoculated with the ACC deaminase-non-producing isolate of *S. violaceorectus* or the control treatment (Figure 5A–C).

Our results indicated that the contents of the photosynthetic pigments, chl *a* and chl *b*, were significantly (*P* < 0.05) higher in *M. chalcea*-inoculated plants than in plants inoculated or not with *S. violaceorectus* at 12 wpt (Figure 5D). The production of carotenoids also was more in plants treated with *M. chalcea*. Similar to the control treatment, *S. violaceorectus*-inoculated plants had lesser contents of total chlorophyll or carotenoids than those inoculated with *M. chalcea* (Figure 5D).

The effect of the two isolates on seed production in *S. bigelovii* was also determined at the time of harvest. In addition to biomass production (Figure 5A–C), *M. chalcea*-treated plants significantly (*P* < 0.05) enhanced seed dry weight by approximately threefold compared to non-inoculated plants (Figure 5E). There were no significant differences in seed production of *S. bigelovii* inoculated with the ACC deaminase-non-producing isolate of *S. violaceorectus* or control treatment. This suggests that *S. bigelovii* can be amenable to improvement by *M. chalcea*.

Together, our results indicate that *M. chalcea* increased plant growth, photosynthetic pigment production and seed yield; whereas *S. violaceorectus* was found to have no significant effect on the growth of *S. bigelovii*.

### Determination of the Endogenous Levels of Auxins and ACC in *S. bigelovii* Tissues

**FIGURE 6:**
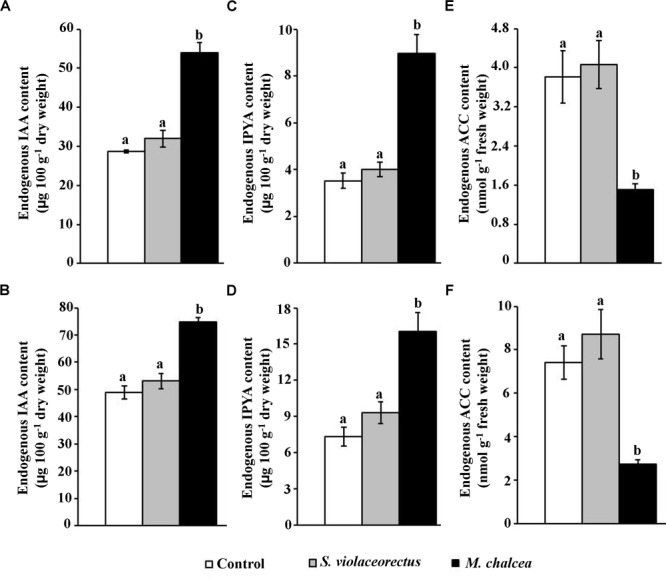
Effect of the application of *Micromonospora chalcea* UAE1 on endogenous auxins and ACC contents of *Salicornia bigelovii* plants. Endogenous auxins and ACC content levels of **(A,B)** IAA; **(C,D)** IPYA; and **(E,F)** ACC upon inoculation with autoclaved starch nitrate broth medium (control), ACC deaminase-non-producing isolate (*S. violaceorectus* UAE1) or ACC deaminase-producing isolate (*M. chalcea* UAE1) of *S. bigelovii* seedlings. Levels of auxins and ACC contents in **(A,C,E)** root and **(B,D,F)** shoot tissues of *S. bigelovii* seedlings grown in an evaporative-cooled greenhouse and maintained at 25 ± 2°C at 12 wpt. Seedlings were inoculated using the pruned-root dip method. Values are means of eight replicates ± SE for each sampling from two independent experiments. Mean values followed by different letters are significantly (*P* < 0.05) different from each other according to Fisher’s Protected LSD Test. IAA, indole-3-acetic acid; IPYA, indole-3-pyruvic acid; ACC, 1-aminocyclopropane-1-carboxylic acid; wpt, weeks post transplantation.

To shed more light on the effect of *M. chalcea* UAE1 in growth promotion of *S. bigelovii*, we measured the endogenous auxin contents and ACC production *in planta*. Only *S. bigelovii* inoculated with the ACC deaminase-producing isolate showed significantly (*P* < 0.05) increased levels of endogenous IAA than the other two treatments in both root (Figure 6A) and shoot (Figure 6B) tissues. We also noted that the auxin IPYA was higher in roots and shoots of *M. chalcea*-inoculated than in *S. violaceorectus*-inoculated and control plants (Figure 6C,D). In this experiment, there was no significant difference between the endogenous levels of IAA and IPYA in the roots and shoots of *S. bigelovii* in control and treated plants with *S. violaceorectus* (Figure 6A–D).

On the other hand, the application of the ACC deaminase-producing isolate (*M. chalcea*) significantly (*P* < 0.05) reduced the ACC levels of in root (Figure 6E) as well as in shoot (Figure 6F) tissues of *S. bigelovii*, compared to the application of the *S. violaceorectus* or the control treatment. In all *in vivo* experiments, there was no significant difference in the endogenous levels of IAA, IPYA, and ACC in the roots and shoots of *S. bigelovii* in control vs. treated plants with *S. violaceorectus* (Figure 6). In all three treatments, the levels of auxins and ACC observed in the shoots were relatively higher than in the roots. Our data suggest that *M. chalcea* is an ACC deaminase-producing isolate that reduces the levels of ACC production to promote the growth of *S. bigelovii*.

## Discussion

In plants, ACC (the natural precursor of the stress hormone ET) plays a crucial role in growth enhancement. Production of ACC deaminase that hydrolyzes ACC to ammonia and α-ketobutyrate, is known to relieve plants from environmental stresses (Glick et al., 2007; Ghosh et al., 2018). High levels of salinity in the UAE’s marine ecosystem is considered as inhospitable environment, and any relief from such stress can be of significance in sustaining better growth performance of *S. bigelovii*. The current research investigated the potential to enhance productivity of *S. bigelovii* growing in nutrient impoverished soils and irrigated with seawater using endophytic PGP actinobacteria capable of producing ACC deaminase. This method can improve the economic viability of *Salicornia* not only as a primary producer, but also as a source of inexpensive biomass for biofuel. Promoting the growth of *Salicornia* spp. using PGPB has widely become an important application in biofuel industries and agricultural activities in the UAE and elsewhere (Bresdin et al., 2016; Sáenz-Mata et al., 2016; Mesa-Marin et al., 2019). Here, we identified and characterized the endophytic actinobacterial isolate, *M. chalcea*, which produced the highest levels of ACC deaminase. This isolate increased the tissue weight and length of *S. bigelovii* plants grown under gnotobiotic and greenhouse conditions irrigated with full strength of seawater compared to the endophytic non-producing ACC deaminase isolate, *S. violaceorectus*. *M. chalcea* was selected based on the production of ACC deaminase and growth-promoting ability under gnotobiotic conditions. Our results demonstrated that *M. chalcea* did not produce any detectable levels of PGRs and was not considered as N-fixer or P-solubilizer. Although many endophytic bacteria were isolated from tissues of *Salicornia* spp. (Jha et al., 2012; Zhao et al., 2016; Yamamoto et al., 2018), this report is the first to reveal the potential of endophytic actinobacteria isolated from the root cortex to promote *Salicornia* growth and to enhance seed yield under greenhouse conditions by possibly lowering the ACC levels *in planta*.

Many terrestrial bacteria including the genera *Enterobacter* (Li et al., 2000), *Bacillus* (Ghosh et al., 2003) and *Streptomyces* spp. (El-Tarabily, 2008) produced ACC deaminase *in vitro*, have been reported to reduce ACC content in plant tissues (Glick et al., 1998) and to promote root elongation and plant growth in horticultural plants. Although marine bacteria isolated from *Salicornia* roots and rhizosphere (Jha et al., 2012; Mapelli et al., 2013), or from mangrove rhizosphere (El-Tarabily and Youssef, 2011) have been known to produce ACC deaminase, the current study is the first to report the production of ACC deaminase from endophytic marine actinobacteria and to determine their effects on growth promotion of halophilic plants, i.e., *S. bigelovii* under greenhouse conditions.

In the present study, the growth promotion by *M. chalcea* was associated with the decrease in the endogenous ACC levels, the enhancement of the photosynthetic pigments and the increase in the endogenous levels of IAA and IPYA in roots and shoots. The increase in seedling growth by *M. chalcea* is similar to other observations where ACC deaminase-producing bacteria enhance plant growth by lowering ET levels (Mayak et al., 2004; El-Tarabily, 2008; El-Tarabily and Youssef, 2011). Following the application of ACC deaminase-producing rhizosphere bacteria, increases in the levels of endogenous auxins in treated plants have also been studied (El-Tarabily, 2008; El-Tarabily and Youssef, 2011). The research under gnotobiotic conditions further established the role of ET as a plant growth inhibitor and the activity of ACC deaminase as a PGP. The use of AVG and ethephon was useful in testing the abilities of the actinobacterial isolates to promote growth of *S. bigelovii*. Applications of AVG, either alone or in combination with actinobacterial isolates, appeared to enhance plant growth by decreasing ACC levels *in planta*. The effects of ethephon in inhibiting growth in *S. bigelovii* can be interpreted as augmentation of ET concentrations in shoot and root tissues. Similar observations using AVG and ethephon have also been found to affect growth in other plant species (Belimov et al., 2001; Ghosh et al., 2003; El-Tarabily, 2008).

*Salicornia* thrive in saline environments that are deficient in N, P, or both (Bashan et al., 2000). Therefore, previous research on PGPB and their effects on *Salicornia* spp. growth predominantly dealt with N-fixing and/or P-solubilizing bacteria. For example, the N-fixing and/or P-solubilizing bacteria obtained from the mangrove rhizosphere affect the growth and performance of *S. bigelovii* irrigated with seawater (Bashan et al., 2000; Rueda-Puente et al., 2003). Under field conditions, inoculation with *Klebsiella pneumoniae* and *Azospirillum halopraeferens*, resulted in increases in *S. bigelovii* growth (weight and length) and biochemical characteristics such as total protein, ash and total lipid content compared to control treatment (Rueda-Puente et al., 2004). Ozawa et al. (2007) have reported that the inoculation of *S. europaea* with the endophytic N-fixing *Pseudomonas pseudoalcaligenes* isolated from *S. europaea* roots under saline conditions resulted in higher concentrations of total N, chlorophyll contents, Na^+^ and K^+^ in the shoot than that of un-inoculated plants. It was also found that the N-fixing endophyte, *Rhodococcus fascians* isolated from *Salicornia* sp., promoted *Salicornia* seed germination and seedling growth under saline conditions in Petri dishes under axenic conditions (Rueda-Puente et al., 2011). Thus, none of these studies were able to consider whether PGPB capable of fixing N or solubilizing P can also promote growth of *Salicornia* spp. through the production of ACC deaminase.

Two reports have investigated bacteria isolated from roots of *Salicornia* spp. that can fix N and produce IAA and ACC deaminase in a chemically defined medium. Yet, these isolates were only tested for *Salicornia* spp. seed germination and seedling performance under axenic conditions in Petri dishes using NaCl (Jha et al., 2012; Zhao et al., 2016). Inoculation with the N-fixing bacteria, *Brachybacterium saurashtrense* and *Pseudomonas* sp. isolated from roots of *Salicornia brachiata*, increased germination percentage, seedling growth and vigor index at elevated NaCl levels (Jha et al., 2012). Interestingly, *B. saurashtrense* and *Pseudomonas* sp. were also found to produce IAA and ACC deaminase, but failed to solubilize P. Under axenic conditions, growth promotion of *S. europaea* seedlings in media supplied with NaCl concentrations, ranging between 50–500 mM, was linked with the ACC deaminase producing endophytic bacteria, *Bacillus* spp., *Planococcus rifietoensis, Variovorax paradoxus* and *Arthrobacter agilis*, isolated from *S. europaea* (Zhao et al., 2016).

Compared with rhizospheric microorganisms, internal colonizers can offer the twin benefits of being acclimated to their hosts, and present at seedling development and rhizosphere initiation (Qin et al., 2015). Bacterial endophytes are indigenous to most plant species, colonizing plant tissues locally or systemically, and both intercellularly and intracellularly (Hallmann et al., 1997; Hardoim et al., 2015; Khare et al., 2018). In the current study, the spores and mycelium of *M. chalcea* were found within the parenchyma cells of the cortex as well as in the xylem. The superior growth promotion of the *M. chalcea* over *S. violaceorectus* was also reflected in the significant reduction of endogenous levels of ACC in root and shoot of *S. bigelovii*. Hence, *M. chalcea* was not considered as a PGR producing, N-fixing or P-solubilizing isolate. The occurrence of *M. chalcea* within the cortical cells of live *S. bigelovii* roots, and the production of ACC deaminase *in situ* within the cortex ensures that the activity of ACC deaminase and possibly other metabolites are produced within the plant roots. Thus, this results in better enhancement of plant growth under stress conditions.

Endophytic actinobacteria are well-known to enhance growth in plants (El-Tarabily et al., 2009; Qin et al., 2015; Trujillo et al., 2015; Vurukonda et al., 2018), eventhough our study is the first to report the response of a *Salicornia* sp. to endophytic actinobacteria under greenhouse conditions. In addition, the root colonization patterns for the ACC deaminase-producing and -non-producing endophytic isolates were tracked for up to 12 wpi. While both *M. chalcea* and *S. violaceorectus* were able to colonize the root tissues, only inoculation with *M. chalcea* resulted in growth promotion. Although *S. violaceorectus* was an endophyte of *S. bigelovii* roots, it was unable to promote growth of *S. bigelovii* under gnotobiotic or greenhouse conditions. This further indicates that the success of *M. chalcea* could be related to its ability to reduce the endogenous levels of ACC inside the roots and shoots of *S. bigelovii*. It is noteworthy that *M. chalcea* and *S. violaceorectus* were incapable of producing detectable levels of IAA, IPYA, GA_3_, iPa, IPA, Z or polyamines *in vitro* (Table 3). This supports the probability that the promotion effect observed in growth of *S. bigelovii* by *M. chalcea* is likely attributed to the activity of ACC deaminase.

In addition to all characteristics discussed above, the potential to use endophytic microorganisms, such as the actinobacteria, relies on their ability to colonize root tissues and to preferentially inhabit these tissues especially in saline, drought and heat environments like those found in the UAE (Vurukonda et al., 2018). Such occupation will also provide the opportunity to *S. bigelovii* and possibly *S. europaea* and other halophytic plants to non-competitively utilize ACC deaminase produced *in situ* by the endophytic isolate. Based on the data obtained, we argue that using microorganisms, including endophytic PGP actinobacteria with ACC deaminase activity, in programs will enhance plant productivity at a field scale in the UAE. It should be noted, however, that growth-promoting factors other than those assayed in the present study may also contribute to the observed PGP. The long term benefit of this approach could include not only the utilization of otherwise non-agricultural land areas boarding the sea in the Arabian Gulf region, but also the profitable venture involving primary production. The outcomes of this investigation are, therefore, expected to develop strategies to utilize saline lands in the UAE for a large scale cropping of halophilic plant species such as *S. bigelovii* for animal feed and for biofuel production purposes. Constructing a mutant of *M. chalcea* UAE1 as a negative control to study the mechanism of PGP is on our priorities for future research.

## Data Availability

The raw data supporting the conclusions of this manuscript will be made available by the authors, without undue reservation, to any qualified researcher.

## Author Contributions

KE-T and SAQ designed and supervised the study, and wrote the manuscript. AAK, LA, and AS performed the greenhouse experiments. AS and KE developed the phylogenetic analyses. KE-T and ST performed the microscopic experiments. KE-T, KE, and SAQ analyzed the data. AAK and MA assisted with the experiments and/or data evaluation. All authors critically revised and approved the final version of the manuscript.

## Conflict of Interest Statement

The authors declare that the research was conducted in the absence of any commercial or financial relationships that could be construed as a potential conflict of interest.
