# Supplementary material for: Growth Promotion of Salicornia bigelovii by Micromonospora chalcea UAE1, an Endophytic 1-Aminocyclopropane-1-Carboxylic Acid Deaminase-Producing Actinobacterial Isolate
Source: Front Microbiol. 2019 Jul 24;10:1694. doi: 10.3389/fmicb.2019.01694 (PMC6668420; doi:10.3389/fmicb.2019.01694)
Supplement: Supplementary file 1 [file Data_Sheet_1.PDF]

Supplementary Figures

Growth promotion of *Salicornia bigelovii* by *Micromonospora chalcea* UAE1, an endophytic 1-aminocyclopropane-1-carboxylic acid deaminase-producing isolate in the United Arab Emirates

Khaled A. El-Tarabily*, Abdulmajeed S. Al Khajeh, Mutamed M. Ayyash, Latifa H. Alnuaimi,

Arjun Sham, Khaled Z. ElBaghdady, Saeed Tariq and Synan F. AbuQamar*

*** Correspondence:**
Dr. Khaled El-Tarabily: ktarabily@uaeu.ac.ae
Dr. Synan AbuQamar: sabuqamar@uaeu.ac.ae

Supplementary Figures

Figure S1. Colonies of endophytic actinobacteria isolated from *Salicornia bigelovii* roots grown on inorganic salt starch agar plates. White arrows represent the streptomycete actinobacterial colonies; whereas red and yellow arrows represent the non-streptomycete actinobacterial colonies of *Actinoplanes* and *Micromonospora* spp., respectively.

Figure S2. Taxonomic determination of *Streptomyces violaceorectus* UAE1, based on phylogenetic, cultural and morphological characteristics. (A) The tree showing the phylogenetic relationships between *S. violaceorectus* UAE1 (*MH255588*; 1,491 bp) and other members of *Streptomyces* spp. on the basis of 16*S* rRNA sequences. (B) Aerial mycelia (left) and substrate mycelia (right) growing on ISP medium 3 supplemented with yeast extract, and (C) scanning electron micrograph (10,000X) of the straight to flexuous (Rectiflexibiles) chains and long, smooth-surfaced spores of the strain of *S. violaceorectus* UAE1. In (A) numbers at nodes indicate percentage levels of bootstrap support based on a maximum likelihood analysis of 1000 resampled datasets. Bar, 0.01 substitutions per site. *S. gardneri* NBRC 3385 (AB184754) was used as an outgroup. GenBank accession numbers are given in parentheses.

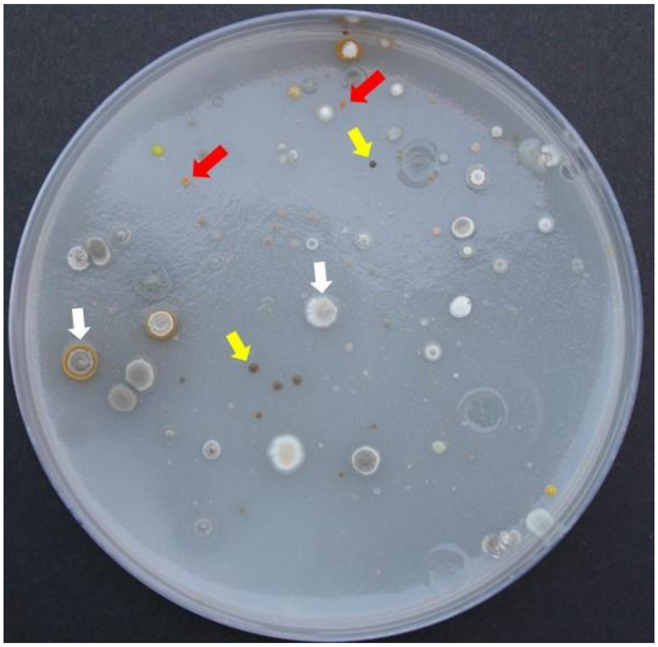

Figure S1. Colonies of endophytic actinobacteria isolated from *Salicornia bigelovii* roots grown on inorganic salt starch agar plates. White arrows represent the streptomycete actinobacterial colonies; whereas red and yellow arrows represent the non-streptomycete actinobacterial colonies of *Actinoplanes* and *Micromonospora* spp., respectively.

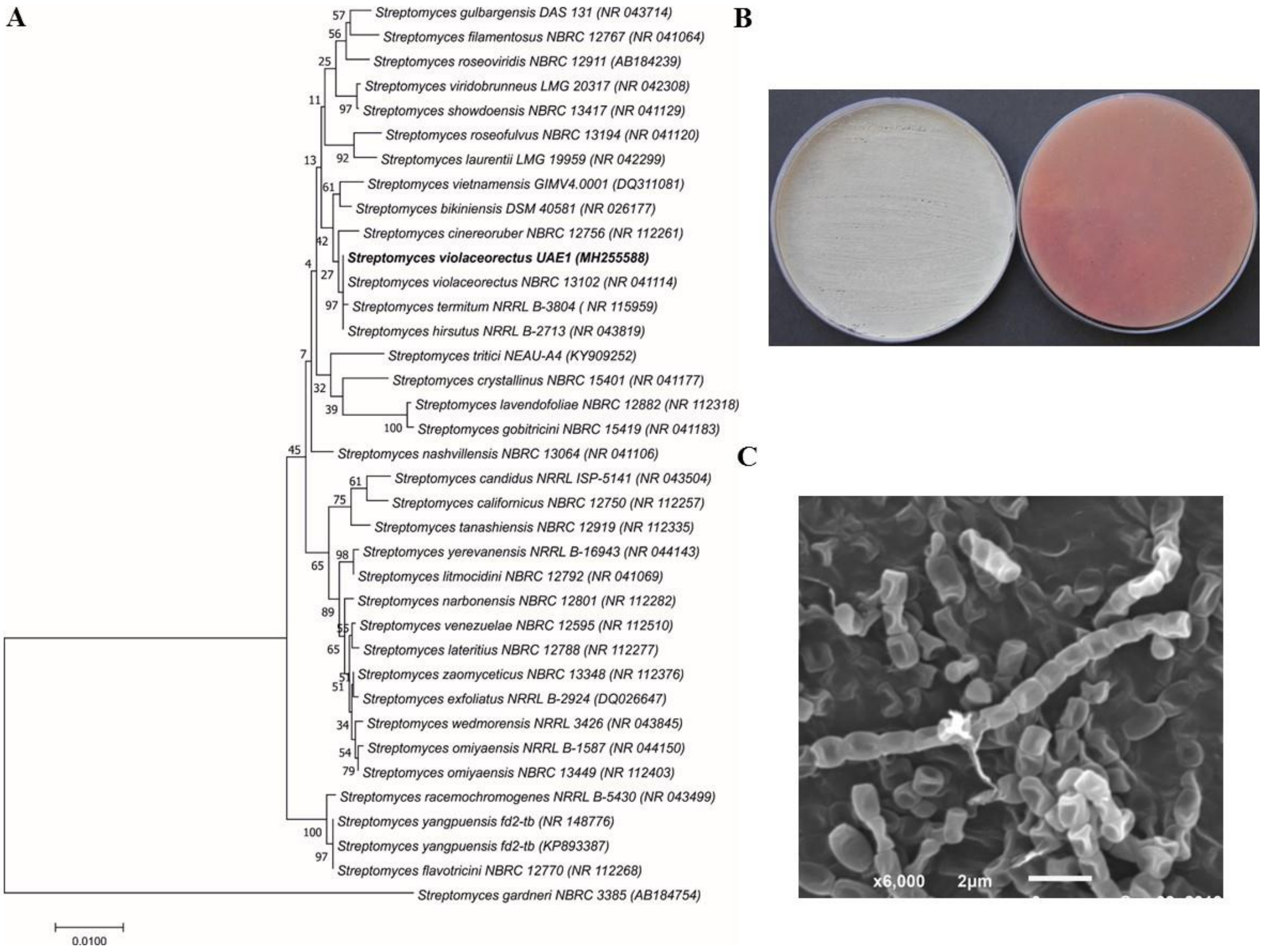

Figure S2. Taxonomic determination of *Streptomyces violaceorectus* UAE1, based on phylogenetic, cultural and morphological characteristics. (A) The tree showing the phylogenetic relationships between *S. violaceorectus* UAE1 (*MH255588*; 1,491 bp) and other members of *Streptomyces* spp. on the basis of 16*S* rRNA sequences. **(B)** Aerial mycelia (left) and substrate mycelia (right) growing on ISP medium 3 supplemented with yeast extract, and **(C)** scanning electron micrograph (10,000X) of the straight to flexuous (Rectiflexibiles) chains and long, smooth-surfaced spores of the strain of *S. violaceorectus* UAE1. In **(A)** numbers at nodes indicate percentage levels of bootstrap support based on a maximum likelihood analysis of 1000 resampled datasets. Bar, 0.01 substitutions per site. *S. gardneri* NBRC 3385 (AB184754) was used as an outgroup. GenBank accession numbers are given in parentheses.
